# Attenuation of neutrophil adhesion and formation of neutrophil extracellular traps by pooled human immune globulins

**DOI:** 10.3389/fphar.2024.1465776

**Published:** 2024-12-12

**Authors:** Vidhya R. Rao, Sana Iqbal, Bradford A. Young, Christine Mun, Sandeep Jain, Simon Kaja

**Affiliations:** ^1^ Department of Ophthalmology, Loyola University Chicago, Maywood, IL, United States; ^2^ Research Service, Edward Hines Jr. Veterans Affairs Hospital, Hines, IL, United States; ^3^ Graduate Program in Molecular Pharmacology and Therapeutics, Loyola University Chicago, Maywood, IL, United States; ^4^ B.A.Y. Biotech Consulting, Annapolis, MD, United States; ^5^ Ophthalmology and Visual Sciences, University of Illinois Chicago, Chicago, IL, United States; ^6^ Selagine, Inc., Chicago, IL, United States; ^7^ Department of Molecular Pharmacology and Neuroscience, Loyola University Chicago, Maywood, IL, United States

**Keywords:** ocular surface disease, dry eye disease, anti-citrullinated protein autoantibodies, neutrophil extracellular trap, pooled human immune globulin

## Abstract

**Introduction:**

This study investigated the efficacy of pooled human immune globulins (Flebogamma^®^ DIF) to combat the formation of neutrophil extracellular traps (NETs) and NETosis, along with neutrophil adhesion to corneal epithelial cells in response to dry eye disease relevant stimuli.

**Methods:**

Human neutrophils were isolated by bead-based immunomagnetic depletion of non-target cells from human whole blood. NETosis was induced using phorbol 12-myristate 13-acetate (PMA) or anti-citrullinated histone 4 R3 antibody (H4R3 ACPA). Extracellular DNA was used as a surrogate biomarker of NETosis, and it was quantified using a 96-well, plate reader-based fluorescent assay and by confocal microscopy in 8-well chambers using the DNA dye, SYTOX^TM^ Green. Neutrophils were labeled with calcein-AM and adhesion to human corneal epithelial cells was measured. The efficacy of a dose-range of pooled human immune globulin (Flebogamma^®^ DIF, 0.01%–5%) was tested in all assays.

**Results:**

Pooled human immune globulins (Flebogamma^®^ DIF) dose-dependently inhibited both PMA and H4R3 ACPA induced NETosis, with concentrations ≥2.5% fully preventing release of extracellular DNA over a 2–16 h time period. Similarly, Flebogamma^®^ 5% DIF prevented NETosis against PMA (20 nM) and a dose range (0.1–10 μg/mL) of H4R3 ACPA. Both PMA and H4R3 ACPA increased adhesion of neutrophils to corneal epithelial cells by 20% and 5%, respectively. Flebogamma^®^ DIF treatment resulted in a dose-dependent reduction of neutrophil adhesion, with Flebogamma^®^ 5% DIF reducing adhesion to baseline levels.

**Discussion:**

These findings show the dose-dependent efficacy of pooled human immune globulins, specifically Flebogamma^®^ DIF against experimentally and pathologically induced NETosis and neutrophil adhesion to corneal epithelial cells, *in vitro*. The results from this study support the continued clinical development of Flebogamma^®^ 5% DIF as a novel and efficacious treatment for the signs and symptoms of dry eye disease.

## Introduction

Dry eye disease (DED) is a multifactorial disease characterized by tear film dyshomeostasis and ocular surface inflammation (Craig et al., 2017a). Patients with DED suffer from ocular irritation, burning, foreign body sensation and visual impairment that severely limits their vision and quality of life (Craig et al., 2017a; Craig et al., 2017b). Chronic inflammation and dysregulated immune system responses contribute to DED pathology (Bron et al., 2017).

Our previous studies have identified extracellular DNA production and dysregulated clearance mechanisms on the ocular surface as significant drivers of DED pathophysiology that was directly corelated to the activation of neutrophils, formation of neutrophil extracellular traps (NETs), and neutrophil death associated with NET formation (NETosis) (An et al., 2019; Kwon et al., 2020; Mun et al., 2019; Sonawane et al., 2012; Tibrewal et al., 2014). NET formation and NETosis are cellular mechanisms, wherein activated neutrophils release extracellular DNA along with neutrophil enzymes such as myeloperoxidase (MPO) and neutrophil elastase that enable the entrapment and killing of pathogens (Thiam et al., 2020). In autoimmune and chronic inflammatory conditions, however, NETosis can cause damage to normal tissues (Thiam et al., 2020). Thus, preventing neutrophil activation and NET formation offers a novel treatment for DED.

We discovered the presence of extracellular DNA (eDNA), neutrophils, and NETs on the ocular surface of DED patients, while nuclease activity in the tear film was significantly reduced (Sonawane et al., 2012). These data suggest that NET accumulation in the precorneal tear film can drive ocular surface inflammation (Sonawane et al., 2012). Indeed, recombinant deoxyribonuclease (DNase) eye drops were effective in reducing the signs and symptoms of DED in patients in a Phase I/II placebo-controlled pilot clinical trial (Mun et al., 2019). Furthermore, hyperosmolar conditions, such as those encountered in evaporative DED result in NETosis, *in vitro*, thus providing a rationale for the excess amounts of neutrophils found on the ocular surface of DED patients (Tibrewal et al., 2014).

More recently, we identified the presence of anti-citrullinated protein autoantibodies (ACPA) in the ocular surface washes from greater than 40% of DED patients, even in the absence of systemic autoimmune disease, with ACPA being absent in washes from healthy eyes (Kwon et al., 2020). These data led us to posit that neutrophil activation and NETosis results in secretion of a peptidyl arginine deiminase 4 (PAD4), the catalytic enzyme responsible for the conversion of arginine to citrulline (also known as citrullination), which can initiate the generation of multiple ACPA species. In support of this hypothesis, ocular surface washes of DED patients elicited NETosis in primary human neutrophils (Kwon et al., 2020), and anti-citrullinated histone 4 R3 antibodies (H4R3 ACPA) elicited DED-like signs in mice, including accumulation of neutrophils and NETs (Kwon et al., 2020). In addition, the inhibition of Fc receptors on neutrophils and dendritic cells as a means to attenuate ACPA-induced effector signaling prevented the DED phenotype in mice.

We, therefore, evaluated the safety and preliminary efficacy of pooled human immune globulins in DED patients in an exploratory Phase 1 clinical trial. Results from this pilot-scale trial showed that pooled human immune globulins delivered at 0.4% (4 mg/mL) twice daily were well-tolerated and significantly reduced signs and symptoms of DED (Kwon et al., 2020).

To determine the most effective dose of pooled human immune globulins for preventing NETosis, we performed a series of *in vitro* experiments in primary human neutrophils. In this study, pooled human immune globulins resulted in a dose-dependent inhibition of extrusion of extracellular DNA from primary human neutrophils and decreased neutrophil adhesion to human corneal epithelial cells in response to pathologically relevant stimuli, supporting the continued clinical development of pooled human immune globulins as a novel treatment for DED.

## Methods

### Isolation of primary human neutrophils

Research was conducted in accordance with the tenets of the Declaration of Helsinki and Institutional Review Board guidelines of Loyola University Chicago. Informed consents were obtained from all participants under the approved protocol, #LU217401. All participants were healthy adult male and female volunteers.

Peripheral blood was collected via venipuncture in vacutainer tubes containing ethylenediamine-tetraacetic acid (EDTA) anti-coagulant (BD Biosciences, Franklin Lakes, NJ).

Human neutrophils were isolated by immunomagnetic depletion of non-target cells using MACSxpress^®^ beads (MACSxpress^®^ Neutrophil Isolation Kit, Miltenyi Biotech, Gaithersburg, MD). Residual erythrocytes were removed using MACSxpress^®^ Erythrocyte Depletion Kit (Miltenyi Biotech). The identity and purity of isolated neutrophils was assessed by flow cytometry (Supplementary Figure S1). Isolated neutrophils were maintained in serum free, phenol red-free RPMI-1640 medium (Gibco, Billings, MT).

### Pooled human immune globulins

In this study, we used Flebogamma^®^ 5% (lot A04H013651) and 10% (lot G04H009631) dual inactivation and filtration development (DIF) (Jorquera, 2009) (Instituto Grifols, S.A, Barcelona, Spain). Flebogamma^®^ DIF is FDA-approved for the treatment of primary (inherited) immunodeficiency and chronic primary immune thrombocytopenia. The U.S. Food and Drug Administration (FDA) considers each pooled human immune globulin preparation a different drug product requiring separate FDA approval and labelling, as physiochemical characteristics and therapeutic efficacy is impacted by methods of manufacturing, sterilization, stabilization and container closure system (Ness, 2019). Therefore, we will refer to the marketed name of Flebogamma^®^ DIF throughout this article to clearly indicate the specific, pooled human immune globulin formulation that was used for these experiments. In addition, we formulated the vehicle based on the approved label for Flebogamma^®^ 5% DIF, comprised of 5% sorbitol, 0.3% polyethylene glycol, pH 5.5, 300 mOsm/L (Vehicle).

### Quantification of NETosis

For microplate-based static and kinetic monitoring of NETosis, neutrophils were seeded at a density of 50,000/0.1 mL media in each well of a poly-d-Lysine (50 μg/mL; Sigma-Aldrich, St. Louis, MO) coated 96-well, clear bottom black plates (Greiner Bio-One, Monroe, NC) and allowed to attach for 30 min. Neutrophils were subsequently stimulated with 20 nM PMA (79346, Sigma-Aldrich) or anti-citrullinated histone 4 R3 antibody (H4R3 ACPA; 1–10,000 ng/mL; Abcam, Waltham, MA, #ab81797). The inhibition of NETosis was determined by conducting the assay in the presence of a dose-range (0.1%–5%) of pooled human immune globulins (Flebogamma^®^ DIF, Instituto Grifols S.A., Barcelona, Spain) or Vehicle.

NETs were monitored by labeling with 1 µM SYTOX^TM^ Green (S7020, Thermo Fisher Scientific, Waltham, MA), a fluorescent dye that is impermeant to live cells. The fluorescence of extracellular NET-bound SYTOX^TM^ Green (Ex/Em λ: 488 nm/510 nm) was measured every 30 min for a period of up to 16 h at 37°C using a microplate reader (Cytation5, BioTek/Agilent, Winooski, VT).

### Confocal microscopy of NETs

Freshly isolated primary human neutrophils were seeded at a density of 100,000/0.2 mL of media in each well of a poly-d-Lysine (50 μg/mL; Sigma Aldrich) coated 8-well chamber slide (Lab-Tek II chamber slide #154534; Thermo Fischer Scientific) and allowed to attach for 30 min at room temperature (RT). Neutrophils were subsequently stimulated with anti-citrullinated histone 4 R3 antibody (H4R3 ACPA; 10–1,000 ng/mL; Abcam, #ab81797) for 16 h or 20 nM PMA (79346, Sigma-Aldrich) for 4 h, in the presence or absence of 0.1%–5% pooled human immune globulins (Flebogamma^®^ DIF) or vehicle. 0.2 mL of 4% paraformaldehyde (PFA) were added directly to each well directly without aspirating the media (final concentration, 2%) and neutrophils and NETs were fixed overnight at 4°C. The following day, chambers were removed, and the slides rinsed with 1× Dulbecco’s modified phosphate buffered saline (DPBS; Corning, Corning, NY). Fixed neutrophils and NETs were blocked with 10% BSA for 1 h and then incubated with a 1:2,000 dilution of DNA labeling dyes SYTOX^TM^ Green (5 mM, #S7020; Invitrogen; Thermo Fischer Scientific) and Hoechst 33342 (20 mM; Thermo Fischer Scientific) diluted in 5% BSA to label extracellular DNA and nuclei, respectively. Following incubation, the slides were rinsed three times in 1× DPBS, air dried, and mounted with coverslip using mounting media (Prolong Diamond Antifade Mount #P36970; Thermo Fischer Scientific). The slides were dried for 24 h prior to imaging. Confocal images of neutrophils and NETs were acquired using a Leica SPE confocal microscope with a ×40 objective and 405 nm and 488 nm laser lines. For analysis, total fluorescence intensity was measured and plotted as normalized fluorescence intensity relative to control (i.e., fold-change).

### Neutrophil adhesion to human corneal epithelial cells

Freshly isolated primary human neutrophils were labelled with 1 µM calcein-AM (#C1430; Thermo Fischer Scientific) for 30 min at RT and subsequently incubated in the presence of a dose-range (0.1%–5%) pooled human immune globulins (Flebogamma^®^ DIF) or vehicle for 30 min at RT. Neutrophils (50,000 cells/90µL/well) were then plated on a confluent layer of human corneal epithelial cells (HCE-T) cells in a 96-well plate. HCE-T cells were obtained from Riken BioResource Research Center (Tokyo, Japan) under Material Transfer Agreement, and maintained as described previously (Ghosh et al., 2021; Araki-Sasaki et al., 1995). Following a 30 min incubation at 37°C in a tissue culture incubator (5% CO_2_/95% humidity), calcein fluorescence was measured to determine the total input fluorescence in each well. Baseline calcein fluorescence was measured using a fluorescence plate reader (Ex/Em λ: 488 nm/510 nm; Cytation5, Biotek/Agilent). Neutrophils were then treated with H4R3 ACPA (100–10,000 ng/mL) or PMA (20 nM) in the continued presence or absence of pooled human immune globulins (Flebogamma^®^ DIF) for 2 h for H4R3 ACPA and 30 min for PMA at 37°C. Non-adherent neutrophils were removed by washing three times with 1× DPBS (0.1 mL). Residual calcein fluorescence was measured after each wash and relative neutrophil adhesion was calculated by dividing fluorescence after the wash by baseline calcein fluorescence, expressed as a percentage.

### Statistical analysis

All analyses were performed using Prism software version 10 (GraphPad Corporation, La Jolla, CA, United Statesa). The results are expressed as mean ± standard error of the mean (s.e.m.). The parametric data were analyzed by One-Way ANOVA followed by Tukey’s or Holm-Šídák multiple comparison test, as appropriate. *P* < 0.05 was considered statistically significant.

## Results

### Pooled human immune globulins (Flebogamma^®^ DIF) dose-dependently attenuate PMA-induced neutrophil activation

**FIGURE 1:**
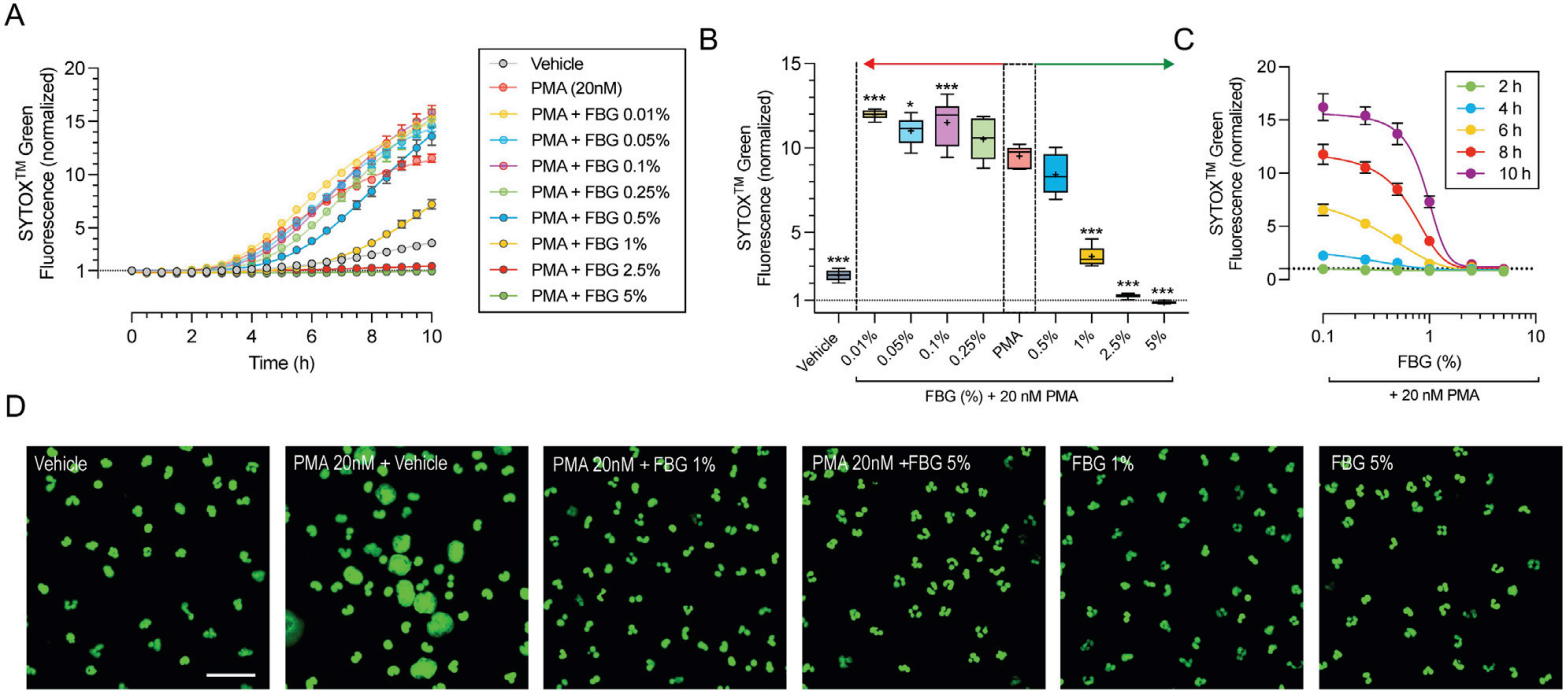
Dose-dependent efficacy of Flebogamma^®^ DIF against PMA-induced NETosis. **(A)** SYTOX^TM^ Green fluorescence normalized to untreated conditions at time 0 vs. time, depicts the time course of NET formation and NETosis of freshly isolated primary human neutrophils in 30 min intervals over a 10 h time period. PMA (20 nM) resulted in a 11.7-fold increase of SYTOX^TM^ Green fluorescence after 10 h. Vehicle elicited a 3.6-fold increase. Pooled human immune globulins (Flebogamma^®^ DIF) prevented PMA-induced increases in SYTOX^TM^ Green fluorescence in a dose-dependent manner. Data were fitted using a non-linear fourth-parameter equation. **(B)** Analysis at 8 h revealed that lower concentrations of Flebogamma^®^ DIF (<0.25%) resulted in modest increases in SYTOX^TM^ Green fluorescence, while higher concentrations (>0.5%) significantly decreased SYTOX^TM^ Green fluorescence. Data were analyzed using One-Way ANOVA (*P* < 0.001) with Dunnett’s multiple comparisons test. *P < 0.05, ***P < 0.001 vs. PMA group. **(C)** Dose-dependency of Flebogamma^®^ DIF at 2, 4, 6, 8, and 10 h. Data were fitted using a non-linear fourth-parameter equation. **(D)** Representative images of inhibition of NET formation and NETosis acquired by confocal microscopy. In fixed cells, SYTOX^TM^ Green labels all nucleic acids and allows visualization of chromatin decondensation, indicative of NETosis, and nuclear morphology. PMA resulted in significant chromatin decondensation associated with NETosis and consistent with the findings of kinetic experiments. Flebogamma^®^ DIF prevented NETosis in primary neutrophils. Normal nuclear morphology was observed in Flebogamma^®^ DIF treated nuclei. Quantification of images is shown in Supplementary Figure S2. Scale bar: 10 µm.

To determine the effect of pooled human immune globulins on NETosis, we exposed freshly isolated primary human neutrophils to PMA (20 nM). The quality and purity of the isolated neutrophils was assessed using flow cytometry by labeling for leukocyte specific cellular markers (Supplementary Figure S1). The levels of extracellular DNA were monitored every 30 min using SYTOX^TM^ Green dye over a period of 10 h (Figure 1A). PMA resulted in a 11.6 ± 0.9 fold (n = 6) increase in SYTOX^TM^ Green fluorescence after 10 h compared to vehicle control, which increased 3.6 ± 0.7 fold (n = 6, *P* < 0.01). To evaluate the effect of Flebogamma^®^ DIF, we analyzed the change in SYTOX^TM^ Green fluorescence after 8 h (Figure 1B). Interestingly, low concentrations of Flebogamma^®^ DIF (<0.25%) resulted in modest increases in SYTOX^TM^ Green fluorescence, while higher concentrations (>0.5%) significantly decreased SYTOX^TM^ Green fluorescence. SYTOX^TM^ Green fluorescence in the presence of 2.5% and 5% Flebogamma^®^ DIF was lower than in the vehicle-treated condition (n = 6, *P* < 0.001; Figure 1B), preventing any increase compared to baseline (i.e., time 0 for the respective condition).

To determine the dose-dependency of Flebogamma^®^ DIF across different timepoints, we plotted normalized SYTOX^TM^ Green fluorescence against the Flebogamma^®^ DIF concentration at 2, 4, 6, 8, and 10 h and fitted the data using a non-linear fourth-parameter equation. Flebogamma^®^ DIF exerted a strong protective effect over this dose-range; R^2^ values for the line of best fit were R^2^ = 0.6 at 2 h, R^2^ = 0.89 at 4 h, R^2^ = 0.92 at 6 h and 8 h and R^2^ = 0.90 at 10 h (n = 6 for each). Notably, 2.5% and 5% Flebogamma^®^ DIF prevented SYTOX^TM^ Green fluorescence at all time points (Figure 1C).

We confirmed the protective effect against NETosis by confocal microscopy following 4 h exposure (Figure 1D). The area fraction (normalized to cell number) increased in the presence of 20 nM PMA to 147.8% (n = 11, *P* < 0.001), while 1% and 5% Flebogamma^®^ DIF reduced the normalized SYTOX^TM^ Green area fraction to 81.4% (n = 12, *P* = 0.64) and 23.8% (n = 5, *P* < 0.001) of Vehicle, respectively (Supplementary Figure S2).

### Pooled human immune globulins (Flebogamma^®^ DIF) dose-dependently mitigate ACPA-induced NETosis

**FIGURE 2:**
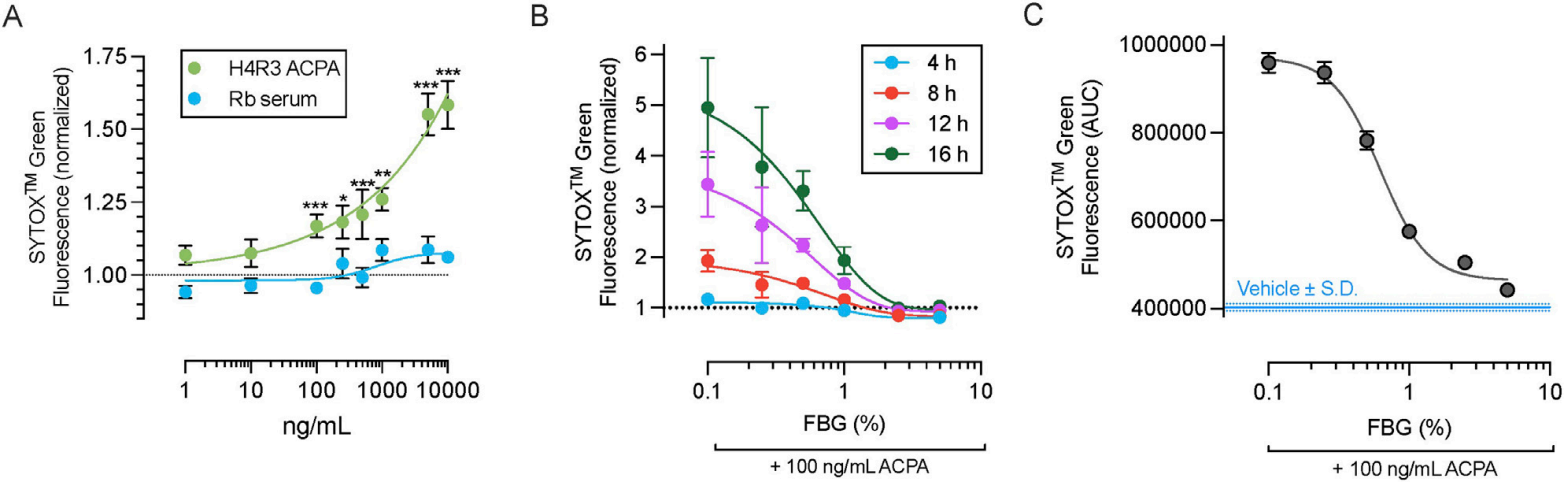
Dose-dependent efficacy of Flebogamma^®^ DIF against H4R3 ACPA-induced NETosis. **(A)** Exposure of neutrophils to H4R3 ACPA for 4 h resulted in significant increase in SYTOX^TM^ Green fluorescence compared to control rabbit serum at concentrations 100 ng/mL or greater. Rabbit serum alone had no significant effect. Data were fitted by non-linear fourth-parameter equation and analyzed using multiple unpaired t-tests with Benjamini, Krieger, and Yekutieli False Discovery Rate (FDR) assessment. *P < 0.05, **P < 0.01, ***P < 0.001 vs. rabbit serum at matched concentration. **(B)** Dose-dependency of Flebogamma^®^ DIF against H4R3 ACPA (100 ng/mL) induced NET formation and NETosis as quantified using SYTOX^TM^ Green fluorescence. Data were fitted using a non-linear fourth-parameter equation. **(C)** Area under the curve (AUC) of SYTOX^TM^ Green fluorescence over a 16 h experiment. Data were fitted using a non-linear fourth-parameter equation. Mean AUC of the Vehicle (402,513 ± 7,807) is indicated by the solid blue line, and standard deviation (S.D.) of the vehicle response is indicated by the dotted blue line.

We have previously demonstrated the pathological role of H4R3 ACPA species in ocular surface disease (Kwon et al., 2020). Thus, we first tested the ability of H4R3 ACPA to induce NETosis using a normal rabbit serum as the concentration-matched control. Exposure of freshly isolated primary human neutrophils to H4R3 ACPA for 4 h resulted in significant increase in SYTOX^TM^ Green fluorescence compared to control rabbit serum at concentrations 100 ng/mL or greater, while rabbit serum alone had no significant effect (Figure 2A).

We selected 100 ng/mL ACPA for subsequent experiments based on these results (described above) and previous data demonstrating the concentration of ACPA in ocular surface washes from patients (Kwon et al., 2020), and experiments showing that this concentration can elicit ocular surface disease in rodents (Kwon et al., 2020; Kaja et al., 2022). Flebogamma^®^ DIF exerted dose-dependent protection against H4R3 ACPA induced NETosis, as assessed at 4 h (R^2^ = 0.64), 8 h (R^2^ = 0.74), 12 h (R^2^ = 0.71), and 16 h (R^2^ = 0.71; n = 3 per timepoint; Figure 2B). This dose-dependency was further demonstrated by calculating the area under the curve (AUC) of SYTOX^TM^ Green fluorescence over 16 h (n = 3, R^2^ = 0.99; Figure 2C). Consistent with our findings for PMA, 2.5% and 5% Flebogamma^®^ DIF completely prevented NETosis over an experimental duration of 16 h.

**FIGURE 3:**
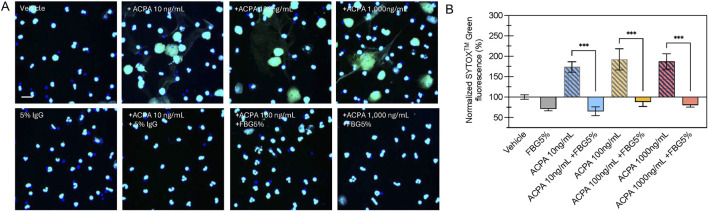
Flebogamma^®^ 5% DIF prevents H4R3 induced NET formation in primary neutrophils. **(A)** Representative confocal microscopy images of NET formation from SYTOX^TM^ Green labeled neutrophils exposed to Vehicle, H4R3 ACPA, Flebogamma^®^ 5% DIF, or H4R3 ACPA + Flebogamma^®^ 5% DIF for 16 h. **(B)** Quantification of NET formation confirmed that pooled human immune globulins (Flebogamma^®^ 5% DIF) completely prevented NET formation. Data were analyzed by One-Way ANOVA (*P* < 0.001) with Tukey’s multiple comparisons test. Select comparisons between H4R3 ACPA in the presence or absence of Flebogamma^®^ DIF are shown. ***P < 0.001. Scale bar: 10 µm.

Confocal microscopy of SYTOX^TM^ Green labeled neutrophils confirmed that H4R3 ACPA elicits NETosis in primary human neutrophils and that 5% Flebogamma^®^ DIF blocked this effect (Figure 3). Specifically, H4R3 ACPA (10–1,000 ng/mL) resulted in significant formation of NETs compared with Vehicle treated neutrophils (n = 6 per group; *P* < 0.001; Figure 3). Pooled human immune globulins (5% Flebogamma^®^ DIF) completely prevented NET formation and integrated density of SYTOX^TM^ Green fluorescence was similar to the Vehicle group following 16 h incubation (n = 6 per group; *P* > 0.05; Figure 3B).

### Pooled human immune globulins (Flebogamma^®^ DIF) dose-dependently inhibit adhesion of neutrophils to corneal epithelial cells

One of the proposed pathophysiological mechanisms underlying neutrophil involvement in DED is the enhanced adhesion of activated neutrophils to the corneal epithelium (An et al., 2019; Kwon et al., 2020). In order test the effect of Flebogamma^®^ DIF on neutrophil adhesion, we labeled freshly isolated primary human neutrophils with calcein-AM, and quantified calcein fluorescence to assess neutrophil adhesion to corneal epithelial cells after 30 min incubation and three washes with DPBS.

**FIGURE 4:**
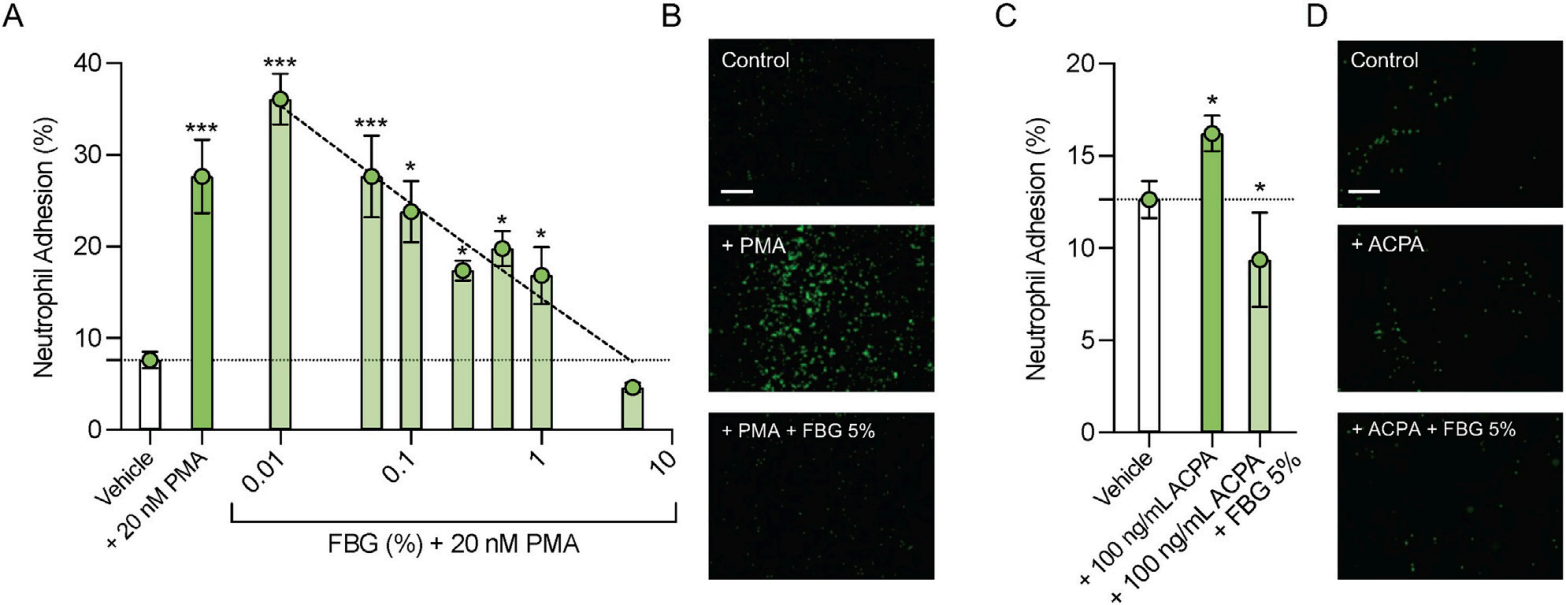
Pooled human immune globulins (Flebogamma^®^ DIF) prevent PMA and H4R3 ACPA induced adhesion to human corneal epithelial cells. **(A)** Exposure to PMA (20 nM) significantly increased neutrophil adhesion to human corneal epithelial cells compared to Vehicle. Flebogamma^®^ DIF dose-dependently reduced adhesion. 5% Flebogamma^®^ DIF completely prevented the PMA mediated increase in adhesion to corneal epithelial cells. Data were analyzed by One-Way ANOVA with Holm-Šídák multiple comparisons test. *P < 0.05, ***P < 0.001 vs. Vehicle. **(B)** Representative low magnification bright field fluorescence microscopy images are shown. **(C)** H4R3 ACPA increased neutrophil adhesion to corneal epithelial cells compared with Vehicle. 5% Flebogamma^®^ DIF reduced adhesion in the presence of H4R3 ACPA to levels below Vehicle. Data were analyzed by Brown-Forsythe ANOVA (*P* < 0.001) with Dunnett’s multiple comparisons test. *P < 0.05 vs. Vehicle. **(D)** Representative low magnification bright field fluorescence microscopy images are shown. Scale bar: 250 µm.

Exposure to PMA (20 nM) increased neutrophil adhesion to corneal epithelial cells from 7.6% ± 0.9% in the Vehicle group to 27.7% ± 4.0% (n = 6, *P* < 0.001; Figure 4A). Pooled human immune globulins dose-dependently reduced adhesion of PMA activated neutrophils to these cells, with 5% Flebogamma^®^ DIF treatment completely preventing the PMA mediated increase in neutrophil adhesion to corneal epithelial cells (4.6% ± 0.5%, n = 6, *P* = 0.48; Figure 4A).

H4R3 ACPA elicited a small, but statistically significant increase in neutrophil adhesion to corneal epithelial cells compared with Vehicle (12.6% ± 0.5% vs. 16.2% ± 0.4%, n = 4–5, *P* < 0.05; Figure 4C). Again, 5% Flebogamma^®^ DIF decreased neutrophil adhesion to lower than Vehicle levels (9.4% ± 1.0%, n = 6, *P* < 0.05; Figure 4C).

## Discussion

Our data demonstrate the dose-dependent efficacy of pooled human immune globulins, specifically Flebogamma^®^ DIF, against experimentally and pathologically induced NET formation and NETosis, *in vitro*. Furthermore, Flebogamma^®^ 5% DIF completely prevented increased neutrophil adhesion to corneal epithelial cells following exposure to PMA- and H4R3 ACPA. As such, these data critically support the ongoing clinical development of Flebogamma^®^ 5% DIF as a novel treatment for the signs and symptoms of dry eye disease (DED).

NETosis on the ocular surface disease is associated with arginine deimination, resulting in the citrullination of histones, specifically H4R3 (Kwon et al., 2020). Arginine deiminase activity is mediated predominantly by PAD4 (Li et al., 2010; Lewis et al., 2015; Martinod et al., 2013), which is secreted by neutrophils and shown to be operative in other organ systems as well (Spengler et al., 2015). Interestingly, our previous findings are consistent with the previous studies that have linked the presence of ACPA to NET formation in rheumatoid arthritis (Wu et al., 2021).

The results from this study extend our previous findings that ACPA-containing ocular surface washes from DED patients could elicit NETosis in primary human neutrophils (Kwon et al., 2020), and that H4R3 ACPA elicited DED-like signs in mice (Kwon et al., 2020). It has previously been demonstrated that histone 4 (H4) by itself induces hydrogen peroxide production and degranulation, as well as increased neutrophil adhesion and pro-inflammatory, IL-8 chemokine secretion (Hsieh et al., 2021). However, to our knowledge this is the first study to describe the kinetics of H4R3 ACPA-induced NETosis and evaluate the adhesion of neutrophils to corneal epithelial cells for this pathology. Notably, H4R3 ACPA resulted in dose-dependent accumulation of extracellular DNA, however, this process was significantly slower and of lower magnitude than that of PMA, and response to H4R3 ACPA showed greater donor variability compared with responses elicited by PMA. The pathologically relevant concentrations of H4R3 ACPA employed only resulted in a two-fold increase in extracellular DNA after 8 h, compared with a twelve-fold increase following PMA. Similarly, neutrophil adhesion to human corneal epithelial cells increased by 5% in response to H4R3 ACPA exposure for 2 h, yet 20% after only a 30 min incubation with PMA. Overall, these findings are consistent with a slowly progressing, chronic, and localized autoimmune phenotype that is typically found in select DED patient populations.

Data presented herein also confirm prior data that have demonstrated the efficacy of pooled human immune globulins against PMA- and calcium ionophore-induced release of extracellular DNA in addition to H4R3 ACPA-elicited NETosis. The exact mechanisms of prevention of extracelluar DNA release by pooled human immune globulins are not known. However, it appears that the protective effects of Flebogamma^®^ DIF are a unique property of pooled human immune globulins vs. single source IgG or protein. It is possible that pooled human immune globulins may, in part, neutralize or sequester PMA and calcium ionophore, however, given the time- and dose-dependent kinetics this is unlikely. Alternatively, pooled human immune globulins may impart Fc-mediated signaling that interferes with ROS generation and intracellular calcium mobilization in neutrophils. In addition to effects on NETosis, pooled human immune globulins may also elicit yet unknown effects on other types of cell death, including apoptosis or necroptosis. Notably, nuclear morphology in PMA-stimulated Flebogamma^®^ DIF-treated human neutrophils was normal and did not show evidence of pyknosis. Our ongoing studies are directed at identification of the detailed molecular mechanisms underlying the efficacy of pooled human immune globulins in preventing release of extracellular DNA from neutrophils stimulated with different insults.

In the present study, we used the SYTOX^TM^ Green DNA dye for quantification of NETosis. Although often considered completely cell-impermeable, there is evidence that SYTOX^TM^ Green flux into cells can give rise to a cell-permeable, non-specific signal (Tatsiy and McDonald, 2018). In order to eliminate the contribution of such non-specific signals in our kinetic assays, the data were normalized to the fluorescence of non-stimulated cells at each respective timepoint. By also measuring NETosis using microscopy, we showed that the fluorescent signal per area was consistent with to that of fluorescence intensity, suggesting that the contribution of the non-specific signal in our experiments was negligible. However, we did note a small effect of Vehicle, especially at later timepoints that may be attributed to an increase in non-specific intracellular SYTOX^TM^ Green fluorescence influx.

Flebogamma^®^ DIF is a pooled, human immune globulin formulation that may help to address the unmet need for better DED treatments and combat this multifactorial disease pathology. Pooled human immune globulins, including Flebogamma^®^ DIF contain naturally occurring antibodies that are protective against pro-inflammatory cytokines and chemokines, and autoantibodies including anti-idiotypic antibodies. The protective heterogeneity of these antibodies increases both with the number of donors and with the concentration of the formulation (von Gunten et al., 2023). In addition, pooled human immune globulins can elicit Fc-dependent effector functions (Kwon et al., 2020). Therefore, higher concentrations of Flebogamma^®^ DIF are likely to show better clinical efficacy using dosing regimens with improved compliance and adherence. To advance the development of Flebogamma^®^ DIF for ocular indications, clinical studies accessing tolerability and dosing are required. Recently, we reported the tolerability and safety of Flebogamma^®^ 5% DIF and Flebogamma^®^ 10% DIF after topical ophthalmic administration in New Zealand White rabbits (Kaja et al., 2024). This present study extends those findings and critically supports the efficacy and dose selection of Flebogamma^®^ 5% DIF against neutrophil-mediated ocular pathology for further development.

Our first-in-human experience with pooled human immune globulins in DED patients stems from an exploratory Phase 1 clinical trial. In this trial, only subjects with ACPA-positive ocular surface washes were included, and subjects were permitted to continue their ocular treatment regimens of prescription and over-the-counter drugs (Kwon et al., 2020). Future explanatory clinical trials in patients with moderate to severe DED will be needed to show whether Flebogamma^®^ 5% DIF has the potential to serve as a first-line treatment for the signs of symptoms of DED.

Different therapeutic modalities have been described to target the pathological consequences of NETosis. In the context of DED, we have previously described the clinical efficacy of DNAse eyedrops (Mun et al., 2019). Interestingly, therapeutic anti-citrullinated protein antibodies (tACPA) have also shown efficacy in a mouse model for lung fibrosis (Chirivi et al., 2021). However, given the unique multimodal benefits of pooled human immune globulins, Flebogamma^®^ DIF has the potential to not only prevent NETosis, but also exert therapeutic efficacy against the non-neutrophil mediated pathology of DED.

In conclusion, pooled human immune globulins demonstrate significant efficacy against NETosis and neutrophil adhesion following ocular surface disease-relevant autoimmune stimuli. As such, these data support the ongoing development of Flebogamma^®^ 5% DIF as novel and efficacious treatment for the signs and symptoms of DED.

## Data Availability

The raw data supporting the conclusions of this article will be made available by the authors, without undue reservation.
