# Supplementary material for: Attenuation of neutrophil adhesion and formation of neutrophil extracellular traps by pooled human immune globulins
Source: Front Pharmacol. 2024 Dec 12;15:1465776. doi: 10.3389/fphar.2024.1465776 (PMC11672200; doi:10.3389/fphar.2024.1465776)
Supplement: Supplementary file 1 [file DataSheet1.pdf]

Supplementary Material

Attenuation of neutrophil adhesion and formation of neutrophil extracellular traps by pooled human immune globulins

Vidhya R. Rao, Sana Iqbal, Bradford A. Young, Christine Mun, Sandeep Jain, and Simon Kaja*

*** Correspondence:**
Simon Kaja, Ph.D.
skaja@luc.edu

1 Supplementary Methods

1.1 Characterization of identity and purity of freshly isolated human neutrophils by flow cytometry

Freshly isolated primary human neutrophils were characterized by flow cytometry using a gating strategy targeting leukocyte specific surface markers with fluorophore conjugated antibodies (Suppl. Table 1).

An isolated population of one million primary human neutrophils were incubated with 0.5 µg of a surface marker antibody in 0.2 mL RPMI media supplemented with 0.5% bovine serum albumin (BSA) for 30 min. Subsequently, neutrophils were washed and centrifuged at 300 × g for 5 min and resuspended in 0.4 mL RPMI media supplemented with 0.5% BSA. For quantification of cell viability, 4′,6-diamidino-2-phenylindole (DAPI) was added at a concentration 0.1 µg/mL. Both surface marker expression and viability were determined by flow cytometry (LSRFortessa™, Beckton, Dickinson and Company, Franklin Lakes, NJ).

2 Supplementary Results

We routinely validate the homogeneity of the freshly isolated primary human neutrophils used in this study by flow cytometry. A representative example of the cell characterization performed using standard flow cytometry procedures and select markers is described here.

The neutrophil gates were first established on unlabeled cells (Suppl. Fig. 1 A-E), and then applied to marker labeled cells (Suppl. Fig. F-K). The side scatter area (SSC-A) *vs*. forward scatter area (FSC-A) analysis identified a live cell population (>98%) suggestive of neutrophils, with the minimal presence of contaminating cell types (Suppl. Fig. 1A,F). The forward scatter height (FSC-H) *vs*.

forward scatter area (FSC-A) gating of the total live cell population (Fig 1 A and F) showed the single cell population (Suppl. Fig. 1B,G). Our neutrophil gating strategy utilized the CD45+ leukocyte cell population (Suppl. Fig. 1C,H). As shown, greater than 95.1% of all cells (and 99.2% of single cells) were $CD45^+$ indicative of a large population of neutrophils (Suppl. Fig. 1H). The homogeneity of our human neutrophil populations was confirmed by co-labeling these cells for CD15 (Sialyl-Lewis^X) and CD16 (FcγRIII), with 93.9% of total cells being $CD45^+CD15^+CD16^+$ (Suppl. Fig. 1I). The total cells were also labeled for CD14 to identify any monocytes and macrophages and CD193 (CCR3) to identify any eosinophils present in our isolated neutrophil samples. As shown, less than 0.2% of total cells were $CD193^+$, while no $CD14^+$ cells were identified (Suppl. Fig. 2K). This representative flow cytometry data shows that our human neutrophil isolation procedure provided a large population of purified neutrophils, as shown by the presence of $CD45^+$ $CD15^+$ $CD16^+$ $CD14^-$ $CD193^-$ cells, with a purity of >95%.

3 Supplementary Figures and Tables

3.1 Supplementary Tables

Supplementary Table 1. List of antibodies

Target	Host	Fluorophore	Manufacturer	Catalog No.
CD14	Monoclonal mouse IgG2aκ	PerCp	Miltenyi Biotec	130-113-712
CD15	Monoclonal mouse IgM	PE	Miltenyi Biotec	130-114-011
CD16	Monoclonal recombinant human IgG1	APC	Miltenyi Biotec	130-113-951
CD45	Monoclonal mouse IgG2aκ	VioBlue	Miltenyi Biotec	130-113-684
CD193	Monoclonal recombinant human IgG1	FITC	Miltenyi Biotec	130-116-662

3.2 Supplementary Figures

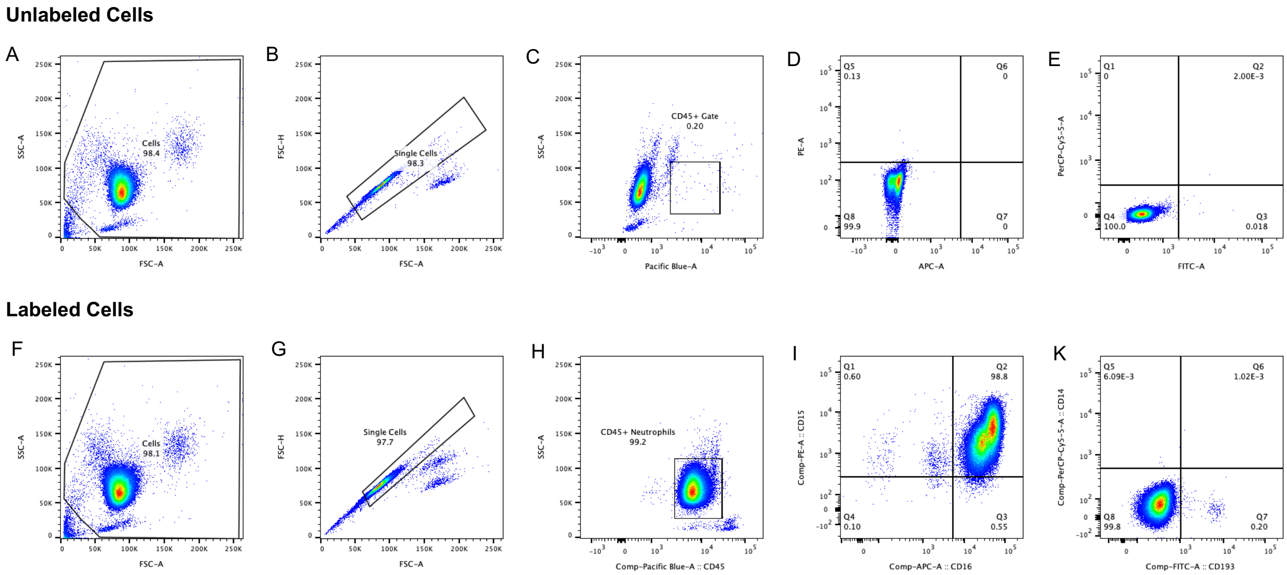

Supplementary Figure 1. Validation of a large population of primary human neutrophils available for subsequent analysis in this study. (**A-E)** Unlabeled cells. **(F-K)** Labeled cells. Cultures present a highly homogenous population of $CD45^{+}CD15^{+}CD16^{+}CD14^{-}CD193^{-}$ neutrophils with >95% purity.

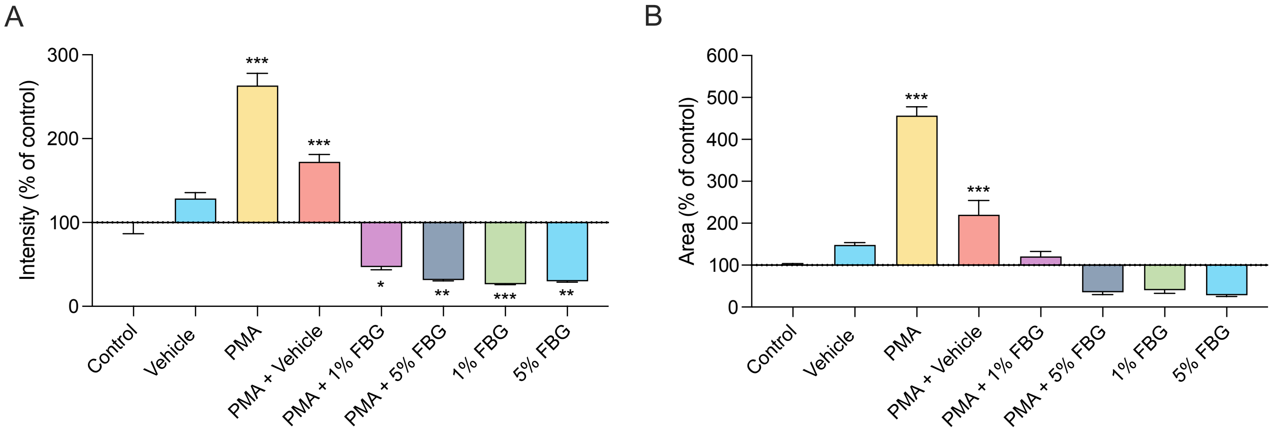

Supplementary Figure 2. Quantification of NETosis acquired by confocal microscopy. (A) Fluorescence was quantified in ImageJ/FiJi software as an integrated density, normalized to the control conditions. Vehicle alone had no significant effect. PMA (20 nM) resulted in a significant increase in fluorescence. Vehicle in the presence of PMA elicited a significant, yet smaller increase in fluorescence. Flebogamma® DIF at 1% and 5% decreased fluorescence in the absence of PMA, and prevented any signal increases in the presence of PMA. **(B)** The effect of pooled human immune globulins was confirmed by quantification of the area fraction of fluorescence, where PMA and PMA + Vehicle resulted in a significant increase that was prevented by both 1% and 5% Flebogamma® DIF. Data were analyzed by One-Way ANOVA ($P < 0.001$) with Dunnett's multiple comparisons test, compared against the Control (Untreated) group. *** $P < 0.001$.
